# Population-based implementation of behavioral health detection and treatment into primary care: early data from New York state

**DOI:** 10.1186/s12913-021-06892-5

**Published:** 2021-09-06

**Authors:** Deborah J. Bowen, Ashley Heald, Erin LePoire, Amy Jones, Danielle Gadbois, Joan Russo, Jay Carruthers

**Affiliations:** 1 Department of Bioethics and Humanities, University of Washington, 1959 NE Pacific Street A204, Seattle, WA 98195 USA; 2 AIMS Center, University of Washington, Seattle, WA USA; 3 New York State, Office of Mental Health, Albany, NY USA; 4 Department of Psychiatry and Behavioral Sciences, Harborview Medical Center University of Washington, Seattle, WA USA; 5 Bureau of Psychiatric Services, NYS Office of Mental Health, New York, USA

**Keywords:** Collaborative Care, Implementation training, Technical assistance, Billing

## Abstract

**Background:**

The Collaborative Care Model is a well-established, evidence-based approach to treating depression and other common behavioral health conditions in primary care settings. Despite a robust evidence base, real world implementation of Collaborative Care has been limited and very slow. The goal of this analysis is to better describe and understand the progression of implementation in the largest state-led Collaborative Care program in the nation—the New York State Collaborative Care Medicaid Program.

Data are presented using the RE-AIM model, examining the proportion of clinics in each of the model’s five steps from 2014 to 2019.

**Methods:**

We used the RE-AIM model to shape our data presentation, focusing on the proportion of clinics moving into each of the five steps of this model over the years of implementation. Data sources included: a New York State Office of Mental Health clinic tracking database, billing applications, quarterly reports, and Medicaid claims.

**Results:**

A total of 84% of clinics with which OMH had an initial contact [*n* = 611clinics (377 FQHCs and 234 non-FQHCs)] received some form of training and technical assistance. Of those, 51% went on to complete a billing application, 41% reported quarterly data at least once, and 20% were able to successfully bill Medicaid. Of clinics that reported data prior to the first quarter of 2019, 79% (*n* = 130) maintained Collaborative Care for 1 year or more. The receipt of any training and technical assistance was significantly associated with our implementation indices: (completed billing application, data reporting, billing Medicaid, and maintaining Collaborative Care). The average percent of patient improvement for depression and anxiety across 155 clinics that had at least one quarter of data was 44.81%. Training and technical assistance source (Office of Mental Health, another source, or both) and intensity (high/low) were significantly related to implementation indices and were observed in FQHC versus non-FQHC samples.

**Conclusions:**

Offering Collaborative Care training and technical assistance, particularly high intensity training and technical assistance, increases the likelihood of implementation. Other state-wide organizations might consider the provision of training and technical assistance when assisting clinics to implement Collaborative Care.

Contributions to the literature

- Research has shown that the Collaborative Care Model is an effective way to treat depression and anxiety within primary care, however real-world implementation continues to be a challenge. Continued training and technical assistance to these clinics may help increase successful implementation.
- Our evidence indicates that high intensity training and technical assistance (more than 10 encounters with a clinic) leads to a higher likelihood of successful implementation.
- These findings contribute to gaps in the literature about how much training and technical assistance is needed per clinic to increase successful implementation, and thus increase the number of primary care clinics offering quality depression and anxiety treatment.
- Combined with previously published literature and tools on the implementation approach New York State used, such as the use of external facilitators, our evaluation findings offer a promising approach other states can utilize to increase behavioral health integration uptake and success.

## Background

The Collaborative Care Model (CoCM) is a leading model for treating mental health issues within primary care. CoCM includes measurement-based treatment to target, population-based perspective, collaboration among clinicians, provision of evidence-based treatments for behavioral health issues, and accountability for patient improvement [1, 2]. Over 80 randomized control trials (RCT) have documented the benefits of CoCM over traditional approaches to treatment of depression, anxiety, and PTSD [3, 4].

CoCM is a team-based approach to treating common behavioral health conditions in a primary care setting. The members of the CoCM team include: the patient, a primary care provider (PCP), a behavioral health care manager (BHCM), and a psychiatric consultant [5]. The PCP oversees all aspects of the patient’s care, including the prescription of all medications [5]. The behavioral health care manager is embedded in the primary care clinic and provides ongoing care coordination and collaboration with the team, including brief, evidence-based behavioral interventions to the patient, as well as routine symptom monitoring and treatment geared toward a measurable target/goal [6]. The psychiatric consultant meets with the BHCM regularly to systematically review cases and make treatment recommendations to the BHCM and PCP [7]. All CoCM patients are tracked in a registry that has the functionality to show treatment processes and outcomes from a population perspective [5]. The BHCM and psychiatric consultant use the registry to aid in clinical decision-making, proactively adjusting treatments that are not working as expected, and ensuring that no patients fall through the cracks.

The NYS Office of Mental Health (OMH), in partnership with the NYS Department of Health (DOH), engaged in a large, state-based program to fully integrate behavioral health screening and treatment into primary care settings, the Collaborative Care Medicaid Program (CCMP) [8]. CCMP grew out of a DOH grant-funded demonstration program from 2011 to 2014. Having demonstrated robust feasibility and acceptability, along with improved clinical outcomes during the grant period, OMH staff were able to secure legislative funding for the creation of the CCMP [8, 9]. CCMP was the first Medicaid program in the country to provide reimbursement for Collaborative Care services. New York submitted a State Plan Amendment which was approved by CMS and provided 50% reimbursement from the federal government.

The unique funding mechanism provided a monthly case rate payment to primary care providers for Medicaid patients receiving CoCM. Reimbursement was originally limited to depression diagnoses for ages 18 and older, and each site had a cap on the number of patients that could be enrolled in order to stay within budget as the program grew. Over time, OMH found that sites were maintaining reasonable caseload sizes and the caps were eliminated. In addition, the age requirement was expanded to ages 12 and over, encouraging more participation from pediatric and family medicine sites. In 2018, based on the feedback from sites about what types of patients they were seeing in their practices, a range of anxiety diagnoses, including Post-Traumatic Stress Disorder, were added to the list of billable diagnosis codes.

In order to ensure fidelity to the evidence-based components of CoCM, clinics interested in billing Medicaid for CoCM services were required to complete a Collaborative Care Medicaid Provider Certification application (billing application), which indicates that the clinic has all of the infrastructure in place for providing CoCM. Requirements include: designating an implementation program lead, a designated psychiatric consultant for weekly, systematic case review of patients, a minimum 0.5 FTE behavioral health care manager with competency in at least one evidence-based intervention for depression and anxiety, a PCP Champion who supports the implementation efforts and can influence other PCPs in the clinic, a billing/data lead, a State-approved registry, and an agreement to submit quarterly metrics data to OMH once CoCM services begin.

Once the application was approved by OMH, the following criteria were met to bill New York State Medicaid each month: a CoCM contact had to be provided, the 9 question Patient Health Questionnaire (PHQ-9) and/or the General Anxiety Disorder 7 Item Scale (GAD-7) had to be administered and recorded, and at least once every 90 days, patients had to be seen face to face in the clinic by either their PCP or the behavioral health care manager for their behavioral health diagnosis. This payment structure allowed care to be provided when and where it is best for the patient’s needs, focusing on outcomes for the patients, instead of being driven by billable encounter criteria.

Another unique feature to the New York State payment structure was the Quality Supplemental Payment (QSP) payment, which gave some clinics the opportunity to get an additional payment by achieving quality outcomes. Primary care practices licensed under Article 28 of the NYS Public Health law, mostly hospital-affiliated outpatient clinics, were eligible for the QSP payments after 3 months of CoCM service if the patient has met one of three quality targets based on the CoCM evidence base. They must either (1), have significantly improved based on a decrease of at least 50% from their baseline PHQ-9 or GAD-7 score, or to a score of below 10. (2) If not improved, have had, a documented change to their treatment plan or (3), their case reviewed by the psychiatric consultant. If any one of these criteria were met, they went on to receive an additional 25% payment for each of those 3 months, retroactively, and each month going forward if one of the quality criteria continued to be met.

The purpose of this paper is to better understand the effects of the state-wide efforts to implement CoCM in primary care practices within New York State. We have used existing clinical and administrative data to document and understand the process of implementation during 2014–2019 in New York, and are optimistic that the project findings will be applicable to other state-based programs as they attempt similar implementations.

## Methods

### Study design

Using a longitudinal observational design, we analyzed existing clinical data from participating CCMP practices to understand the process of CoCM implementation from 2014 to 2019. We used the Reach, Effectiveness, Adoption, Implementation, and Maintenance (RE-AIM) model, a well-established framework for translating research into real-world implementation of evidence-based interventions to frame our data presentation, focusing on proportion of clinics moving into each of the five steps of this model over the years of implementation [10]. Data sources included: an OMH clinic tracking database, Medicaid billing applications, quarterly reports of performance and outcomes, and Medicaid claims. The estimated number of primary clinics of all types is 9548, across the state. To arrive at this number, we used the New York State Department of Health’s Provider Network Data System to see how many individual providers submitted Medicaid claims as of December 2019 [11]. Of those, we sorted by provider address and eliminated duplicates, which gave us the number of clinics in New York State submitting Medicaid claims for primary care services. We filtered out clinics for which family medicine, internal medicine, or pediatrics were not the primary specialty of the clinic.

All sites that participated in CCMP had access to training and technical assistance (TTA) during CoCM implementation. Common TTA activities include individual and group phone calls, webinars, full and half day in person trainings, and online modules. Topics commonly covered in TTA include billing, hiring, documenting, clinical skills, and many other topics tailored to a specific clinic’s needs. OMH staff collaborated with the Advancing Integrated Mental Health Solutions (AIMS) Center to provide evidence-based implementation plans and processes for all participating primary care settings implementing CoCM. Since 2014, OMH has offered continuous CoCM implementation TTA, free of charge, to all primary care clinics interested in participating in the CCMP, as well as ongoing TTA after clinics begin offering these services to their patients. Data were collected since CCMP began.

### Implementation model

OMH partnered with the AIMS Center at the University of Washington to provide TTA for the CCMP. The AIMS center follows a 5- step implementation process: 1) Lay the Foundation, 2) Plan for Clinical Practice Change, 3) Build Your Clinical Skills, 4) Launch Your Care, and 5) Nurture Your Care [12]. Step one focuses on education and orientation to the model and the importance of organizational leadership support. Step two involves creating an implementation plan and identification of care team members within the site, often using coaching calls and online training modules to get team members ready to implement. Step 3 focuses on clinical training, often through online and in-person training sessions that providers attend followed with topical webinars to support use of the model. OMH offered free and/or discounted Problem-Solving Treatment (PST) certification training through the AIMS Center. PST is an evidence-based behavioral health intervention particularly well-suited to primary care settings [13]. Step 4 is launching the implementation and using the registry to track patients and ensure quality of CoCM. OMH commissioned the AIMS Center to build a customized behavioral health registry program, which was a version of the Care Management Tracking System (CMTS), and contained process and outcomes metrics specific to the Collaborative Care Medicaid Program [14]. CMTS is a web-based registry used for systematically tracking behavioral health caseloads and generating reports to facilitate clinical decision-making and quality improvement. OMH offered clinics access to this version of CMTS at no cost for 1 year and then at an extremely discounted rate after that. Clinics providing CoCM were required to use some type of registry approved by OMH in order to bill the New York State Medicaid codes and the CoCM CPT codes for other payers, but were not required to use CMTS specifically. Ongoing coaching calls, case presentations, and monthly office hours were used to support sites through both steps four and five, which focused on sustainment of the model.

TTA was further enhanced through the utilization of a New York-based AIMS Center external facilitator and her team of implementation specialists who, in addition to conducting most of the activities listed above, provided site visits and specialized expertise in New York State licensure and billing laws. In addition, a website was created specifically for CCMP that housed many resources, including a TTA calendar and recordings of webinars. While TTA was offered to all participants, it was not a requirement of participation. Each clinic was free to decide how much or how little assistance they consumed, or whether they consumed any assistance at all. TTA was meant to help clinics get the infrastructure in place and provide on-going support once services began. Some clinics chose to get their TTA through sources other than or in addition to OMH including, but not limited to, Delivery System Reform Incentive Payment (DSRIP) Preferred Provider Systems, New York City Department of Health and Mental Hygiene (NYC DOHMH) Mental Health Service Corps, and other national content experts such as the National Council for Behavioral Health and the American Psychiatric Association.

### Measurements

We included recruitment and participation records data from primary care clinics located in New York State that expressed an interest in participating in the Collaborative Care Medicaid Program (CCMP) between 2014 and 2019. Clinics that participated in the Collaborative Care Initiative from 2012 to 2014, were grandfathered into this data set. We defined each of the following RE-AIM framework steps as:

Reach, ever contacting OMH with interest in CoCM. In our analyses, expression of interest was indicated through an initial contact between OMH and an individual clinic or group of clinics. These contacts occurred over the telephone and in person, with OMH providing information about the requirements for participation in the CCMP, along with a CCMP billing application, followed by a discussion to determine the feasibility of the clinic meeting those requirements with or without TTA from OMH or outside sources. The requirements for participation are stated in the CCMP billing application [15]. In some cases, organization leaders met with OMH and let individual sites within their organization decide whether to participate. If an organization met with OMH, we marked all clinics under that organization as having an initial contact.

Effectiveness, how well the program worked in achieving clinical goals of lowering depression and anxiety levels in patients. Using the quarterly reports OMH collected from participating clinics, we were able to determine an average depression and anxiety improvement rate. Improvement was defined by OMH in 2018–2019 as “the number and proportion of patients enrolled in treatment for 70 days or greater who demonstrated clinically significant improvement either by (1), a 50% reduction from baseline PHQ-9/GAD-7 or (2), a drop from PHQ-9/GAD-7 to less than 10” [16].

Adoption, whether clinics received any training and technical assistance. Consumption of TTA was not mandatory and varied greatly among clinics. Adoption was further defined by who provided the TTA (OMH only, an outside source only, or both OMH and an outside source) and level of TTA intensity (high if over 10 encounters with a clinic, low if under 10 encounters with a clinic, and unknown if the intensity could not be determined).The cutoff of 10 TTA encounters as being indicative of high intensity was determined after consultation with a number of TTA providers, based on their collective experience working with clinics.

Variables for implementation, which is the extent to which the intervention is implemented as intended in the real world, were defined as: 1) the completion of a CCMP billing application, which attested that all of the components of CoCM were in place 2) the submission of at least one quarter of data to OMH. A clinic’s submission of quarterly metrics data indicates that it has all the key components in place *and* is currently providing CoCM services or 3) the submission of Medicaid billing claims. Developing a workflow to bill Medicaid can be quite challenging for clinics, so we considered this to be the most extensive measure of implementation.

Finally, we looked at maintenance of clinics’ CoCM programs long-term, defined as submitting at least one quarterly report 1 year or more since their first quarterly report submission.

The FQHC dataset consisted of all New York State grantees of the Health Resources and Services Administration (HRSA)‘s 2018, Health Center Awardee’s under the Unified Data System that met our inclusion criteria of providing primary care to patients 12 years and older [17]. Clinics were excluded if they were homeless shelters, school-based health clinics, church-affiliated services, dental clinics, mobile clinics, and administrative-only offices. A total of 452 FQHC clinics were included in our analyses. OMH kept a record of every FQHC and non-FQHC with which it had contact in its clinic tracking database (*n* = 611), as well as TTA source and intensity data.

### Data analysis

Independent T-tests were run to determine significance of relationship between steps of RE-AIM and reception of and intensity of TTA. We performed the t- tests on the following variables: receipt of TTA and completion of a billing application, receipt of any TTA and reporting at least one quarter of data, receipt of any TTA and billing Medicaid, receipt of any TTA and reporting quarterly data at least 1 year after their first report. Other analyses include looking at type of TTA received (from OMH, from another source, or a combination) and how that related to implementation and maintenance outcomes. Specific variables included: type of TTA and completion of billing application, type of TTA and reporting at least on quarter of data, type of TTA and billing for Medicaid, and type of TTA and reporting quarterly data at least 1 year after the first quarterly report. Analyses were also conducted on intensity (high, low, unknown) of TTA and our implementation and maintenance variables. These include: TTA intensity and completion of billing application, TTA intensity and reporting at least one quarter of data, TTA intensity and billing Medicaid, and TTA intensity and reporting data at least 1 year after first quarter report. Finally, we categorized clinics into effective (overall improvement greater than or equal to 33%) or not and analyzed effectiveness to both TTA intensity and TTA source. All data were analyzed using SPSS version 27 [18].

## Results

### Analytic sample formation

Clinics analyzed included a combination of the HRSA FQHC list and OMH’s tracking database (*n* = 891). We excluded clinics from the HRSA FQHC list that are not considered primary care clinics, such as homeless shelters, school-based health clinics, church-affiliated services, dental clinics, mobile clinics, and administrative-only offices. After removing these clinics, we were left with 452 FQHCs, of which 377 had contact with OMH.

Table 1 shows the background data collected as part of HRSA’s Health Center Program Awardee program [17]. As seen in this table, FQHCs serve a diverse and large number of patients across the state. FQHC’s had relatively high numbers of Medicaid and uninsured patients (a total of 70%), and a relatively large number of non-White patients (29%). The safety net nature of FQHCs has resulted in a defined population of underserved patients in the sample.

**Table 1:** Background data for eligible FQHCs in the present study

	Average (and standard deviation) across the 452 Eligible FQHC’s only
Average number of patients in clinic organizations	88,609 (73,650.7)
Percentage of racial/ethnic minority patients in the clinics	71.57% (29.63%)
Percent of patients aged over 65 years in the clinics	9.06% (4.14%)
Percent of patients best served in a language other than English in the clinics	29.19% (20.66%)
Payer mix (percentages) per clinic	
Medicare	10.15% (4.97%)
Medicaid	54.20% (11.62%)
Uninsured	16.41% (10.23%)
Third Party Insurance	19.24% (10.09%)
Percent of mental health patients per clinic	9.91% (10.51%)
Percent screened for clinical depression who scored positive and had a documented follow-up plan per clinic	74.08% (17.56%)

**Fig. 1:**
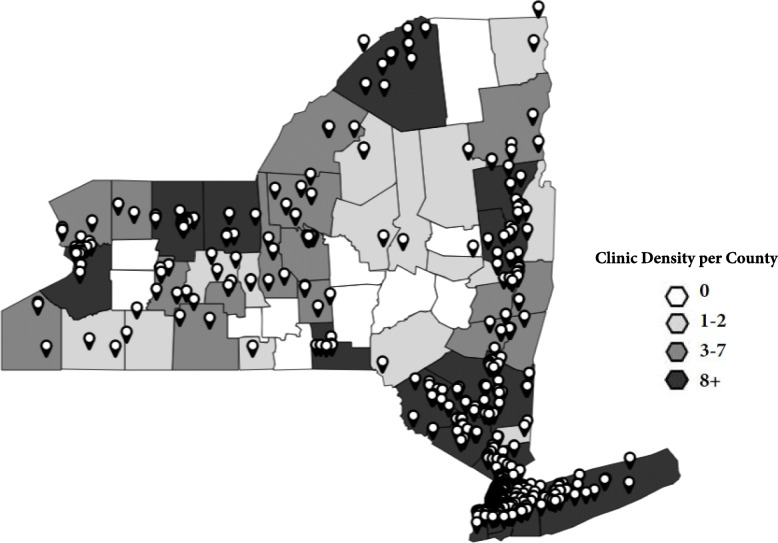
Map of the reach of this project in New York State between 2014 and 2019, based on density per county (n clinics = 611). Map of New York State with outlines of each County. Counties have one of 4 shades indicating the density of clinics included in this study per county, with white indicating 0 clinics, light grey 1–2 clinics, medium grey 5–7 clinics and dark grey 8 or more clinics. Pins indicate the exact location of clinics included in the study on the map. The map was created by investigators using Mapline

**Fig. 2:**
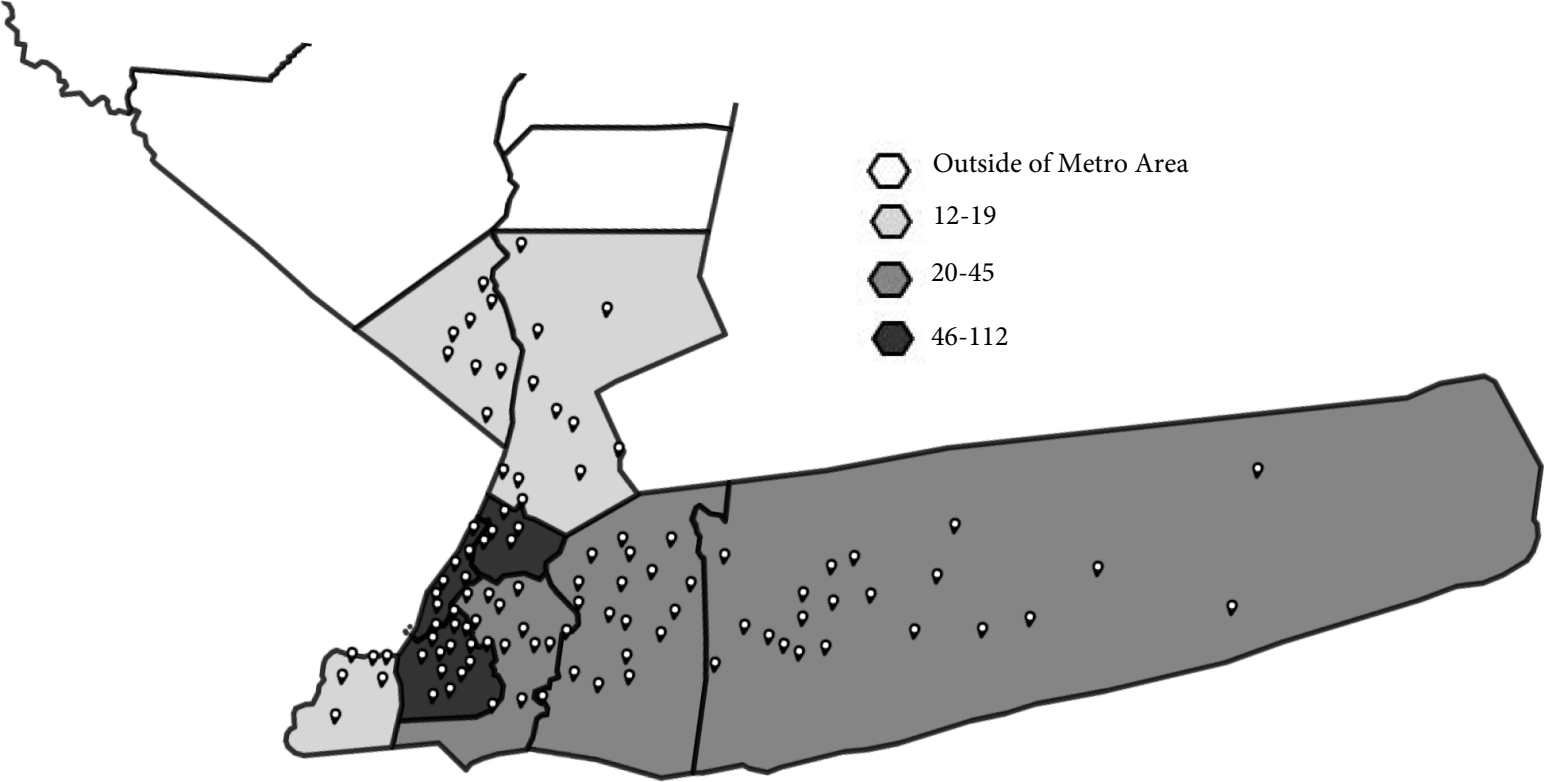
Map of the reach of this project in the New York City metropolitan area (*n* = 426). A zoomed in portion of Fig. 1, showcasing clinic density per county in the New York City Metropolitan Area. Counties have one of 4 shades indicating the density of clinics included in this study per county, with white indicating the county is outside of the metropolitan area, light grey 12–19 clinics, medium grey 20–45 clinics, and dark grey 46–112 clinics. Pins indicate the exact location of clinics included in the study on the map. The map was created by investigators using Mapline

Figure 1 presents a map of New York State, with clinics in the reach phase marked by symbols. As previously stated, we defined reach as the number of clinics with which OMH had contact out of all primary care clinics in New York State. While the reach of this program is low (6%), we believe a visual representation of the clinics we did reach is valuable to illustrate the breakdown of reach by county. Figure 2 is a close up of the New York City metropolitan area. Population by county is available in Table 2.

**Table 2:** New York State Population by County

County	2019 Population	Clinics that ever had contact with OMH (Reach)
Albany	**305,506**	**4**
Allegany	**46,091**	**2**
Bronx	**1,418,207**	**92**
Broome	**190,488**	**6**
Cattaragus	**76,117**	**2**
Cayuga	**76,576**	**3**
Chautauqua	**126,903**	**3**
Chemung	**83,456**	**1**
Chenango	**47,207**	**0**
Clinton	**80,485**	**2**
Columbia	**59,461**	**6**
Cortland	**47,581**	**4**
Delaware	**44,135**	**1**
Dutchess	**294,218**	**8**
Erie	**918,702**	**11**
Essex	**36,885**	**4**
Franklin	**50,022**	**0**
Fulton	**53,383**	**0**
Genesee	**57,280**	**0**
Greene	**47,188**	**5**
Hamilton	**4416**	**1**
Herkimer	**61,319**	**1**
Jefferson	**109,834**	**4**
Kings	**2,559,903**	**112**
Lewis	**26,296**	**2**
Livingston	**62,914**	**4**
Madison	**70,941**	**0**
Monroe	**741,770**	**23**
Montgomery	**49,221**	**1**
Nassau	**1,356,924**	**21**
New York	**1,628,706**	**74**
Niagara	**209,281**	**4**
Oneida	**228,671**	**1**
Onondaga	**460,528**	**5**
Ontario	**109,777**	**2**
Orange	**384,940**	**17**
Orleans	**40,352**	**2**
Oswego	**117,124**	**5**
Otsego	**59,493**	**0**
Putnam	**98,320**	**2**
Queens	**2,253,858**	**45**
Rensselaer	**158,714**	**7**
Richmond	**476,143**	**13**
Rockland	**325,789**	**9**
St. Lawrence	**107,740**	**7**
Saratoga	**229,863**	**8**
Schenectady	**155,299**	**2**
Schoharie	**30,999**	**0**
Schuyler	**17,807**	**0**
Seneca	**34,016**	**2**
Steuben	**95,379**	**4**
Suffolk	**1,476,601**	**29**
Sullivan	**75,432**	**8**
Tioga	**48,203**	**0**
Tompkins	**102,180**	**0**
Ulster	**177,573**	**10**
Warren	**63,944**	**8**
Washington	**61,204**	**1**
Wayne	**89,918**	**5**
Westchester	**967,506**	**14**
Wyoming	**39,859**	**0**
Yates	**24,913**	**4**

**Fig. 3:**
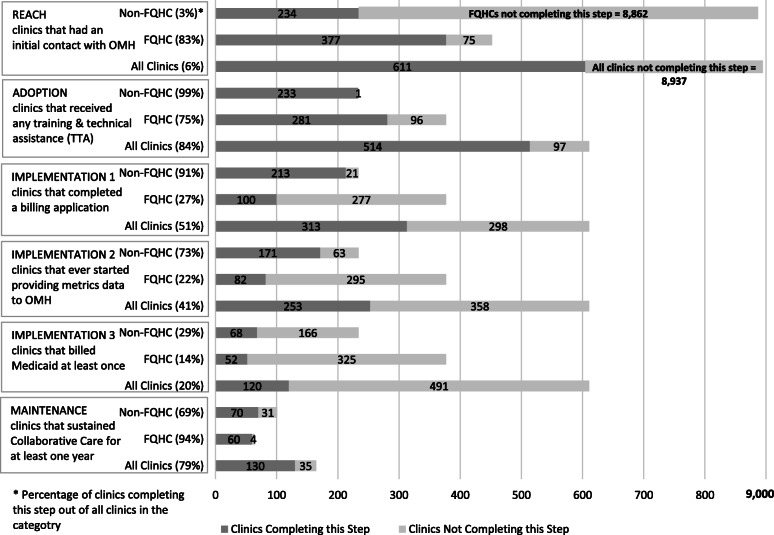
RE-AIM Steps and Definitions by Clinic Type. Number (and %) of FQHCs, non-FQHC’s, and total number of clinics completing each RE-AIM step. Dark grey lines indicate the number of clinics that completed the step. Light grey indicates the number of clinics not completing the step. Created in Microsoft Excel by investigators

Fig. 3 presents the RE-AIM model phases achieved by clinics across the state of New York during the study period. Each phase of the RE-AIM model is addressed in this figure.

### Reach

OMH reached 611 clinics out of 9548 primary care clinics in New York State (6%). Of these clinics, 377 were FQHCs, (83% of all eligible FQHCs), and 234 were general primary care clinics (3%).

### Effectiveness

A majority of clinics that attempted to implement CoCM achieved clinically significant improvement in their patients’ depression and/or anxiety symptoms, as indicated through an examination of annual data reports of PHQ-9 and GAD-7 scores for the years 2018–2019. The average percent of improvement across 155 clinics that submitted at least one quarter of improvement data between 2018 and 2019 was 44.81%. An independent t-test was run using SPSS statistical software in order to determine if there was a significant difference in improvement between TTA source groups and TTA intensity groups, but no significant differences were found in improvement by TTA source or intensity (*p* = .144, *p* = .561 respectively).

### Adoption

Overall, a high percentage of clinics used the assistance of OMH and/or other sources to implement CoCM during the implementation period. Of clinics that OMH reached, 75% of received some form of TTA, with 61% of those receiving TTA from OMH only, 10% receiving TTA from another source, and 29% receiving TTA from both OMH and another source. Intensity of TTA was split relatively evenly among clinics with 44% receiving high intensity TTA, 51% receiving low intensity TTA, and 5% received an unknown amount of TTA.

### Implementation

There were multiple variables used for the implementation analyses in this study. As seen in Fig. 3, we selected three that had the most relevance to the implementation process for clinics. The first was the number of clinics that completed and submitted a CCMP billing application. A total of 47% of clinics reached by OMH went on to complete a CCMP billing application. As seen in Table 3, clinics that received TTA from OMH and/or received high intensity TTA were significantly more likely to complete a billing application (*P* < .001).

**Table 3:** Relationship of Technical assistance receipt and implementation of protocols for CoCM in New York State

FQHC’s Only (*N* = 377)					Non- FQHC’s (*n* = 234)				
Technical Assistance Status	Implementation- completed a billing application (*n* = 100)	Implementation- Reporting Outcomes (*n* = 82)	Maintenance (*n* = 60)	Implementation- Billed Medicaid (*n* = 52)	Technical Assistance Status	Implementation- Application for Billing (*n* = 213)	Implementation- Reporting Outcomes (*n* = 171)	Maintenance (*n* = 70)	Implementation- Billed Medicaid (*n* = 68)
Received TA (*n* = 281)	93^a^ (93%)	82^a^ (100%)	60^a^ (100%)	51^a^ (98%)	Received TA (*n* = 233)	213 (100%)	171 (100%)	70 (100%)	68 (100%)
Never received TA (*n* = 96)	7 (7%)	0 (0%)	0 (0%)	1 (2%)	Never received TA (*n =* 1)	0 (0%)	0 (0%)	0 (0%)	0 (0%)
Intensity of Technical Assistance	Implementation- Application for Billing (*n =* 100)	Implementation- Reporting Outcomes (*n =* 82)	Maintenance (*n =* 60)	Implementation- Billed Medicaid (*n =* 52)	Intensity of Technical Assistance	Implementation- Application for Billing (*n =* 213)	Implementation- Reporting Outcomes (*n =* 171)	Maintenance (*n =* 70)	Implementation- Billed Medicaid (*n =* 68)
High intensity (*n =* 99)	73^a^ (73%)	66^a^ (80%)	48^a^ (80%)	43^a^ (83%)	High intensity (*n* = 126)	126^a^ (59%)	112^a^ (65%)	50^a^ (71%)	45 (66%)
Low intensity (*n* = 158)	20 (20%)	16 (20%)	12 (20%)	8 (15%)	Low intensity (*n* = 106)	87 (41%)	59 (35%)	20 (29%)	23 (34%)
Unknown intensity (*n* = 24)	0 (0%)	0 (0%)	0 (0%)	0 (0%)	Unknown intensity (*n =* 1)	0 (0%)	0 (0%)	0 (0%)	0 (0%)
Never Received TA (*n =* 96)	7 (7%)	0 (0%)	0 (0%)	1 (2%)	Never Received TA (*n =* 1)	0 (0%)	0 (0%)	0 (0%)	0 (0%)
Technical Assistance Source	Implementation- Application for Billing (*n =* 100)	Implementation- Reporting Outcomes (*n =* 82)	Maintenance (*n =* 60)	Implementation- Billed Medicaid (*n =* 52)	Technical Assistance Source	Implementation- Application for Billing (*n =* 213)	Implementation- Reporting Outcomes (*n =* 171)	Maintenance (*n =* 70)	Implementation- Billed Medicaid (*n =* 68)
Received TA from OMH only (*n =* 202)	49^a^ (49%)	47^a^ (57%)	32^a^ (53%)	26^a^ (50%)	Received TA from OMH only (*n* = 112)	95^a^ (44%)	72^a^ (43%)	19^a^ (28%)	28^a^ (41%)
Received TA from Another Source Only (*n* = 22)	0 (0%)	0 (0%)	0 (0%)	0 (0%)	Received TA from Another Source Only (*n =* 28)	25 (12%)	23 (13%)	8 (12%)	1 (1%)
Received TA from Both OMH and Another Source (*n* = 57)	44 (44%)	35 (43%)	28 (48%)	25 (48%)	Received TA from Both OMH and Another Source (*n* = 93)	93 (44%)	76 (44%)	42 (60%)	39 (58%)
Never Received TA (*n =* 96)	7 (7%)	0 (0%)	0 (0%)	1 (2%)	Never Received TA (=1)	0 (0%)	0 (0%)	0 (0%)	0 (0%)

^a^indicates a significance level of <.001

The second phase of implementation was the number of clinics that reported process and outcomes data. Of clinics OMH reached, 37% submitted at least one quarterly report. Of the clinics that submitted a billing application, a total of 81% achieved this standard. Clinics that received TTA from OMH and/or high intensity TTA were significantly more likely to submit at least one quarterly report (*p* < .001).

The final phase of implementation was the number of clinics that successfully billed the CoCM Medicaid case rate. Of all clinics that OMH reached, 17% were able to successfully bill Medicaid for CoCM, with FQHC’s significantly more likely to bill Medicaid compared with other primary care clinics. Of clinics that completed a billing application, 38% (*p <* .001) of clinics that completed a billing application and 47% of clinics that submitted at least one quarter of metrics data went on to successfully bill Medicaid.

### Maintenance

We defined maintenance as the number of clinics that were able to sustain CoCM for at least 1 year after their first quarterly report was submitted. A total of 79% (*n* = 130) of clinics that reported data before the first quarter of 2019 sustained CoCM for 1 year or more.

Role of training and technical assistance in implementation.

Further exploration of the role of training and technical assistance in implementation can be seen in Table 3. It is clear from the data in this table that receipt of TTA is associated with successful implementation, as measured by all three of our markers of implementation. For both FQHCs and all clinics, TTA receipt versus no TTA receipt was associated significantly with our three implementation indices, and intensity of TTA appeared to be linearly related to implementation, such that clinics receiving more intensive TTA reported better implementation than did clinics receiving low intensity TTA (all ps significant at <.001). The source of TTA made a consistent difference in implementation, as clinics receiving TTA from OMH only reported better implementation than clinics receiving TTA from another source.

## Discussion

The purpose of this study was to describe and understand the progress toward statewide implementation of Collaborative Care in a large state-sponsored initiative, the New York State Collaborative Care Medicaid Program. This paper describes progress toward that goal, using the RE-AIM model as a useful organizing framework for looking at population-based data on implementation over time in this project. As with many applications of the RE-AIM model to real life problems, the penetrance achieved is directly related to the methods of calculation of the proportion of clinics making it to the next step. While only 6% of all primary care clinics were reached by OMH, the percentage of clinics that went on to bill for CoCM from among those that were reached was 20%. Those practices that were successfully reached had good rates of adoption and implementation. Despite the strong evidence base supporting the model for over two decades, CoCM has seen slow diffusion. So, although the percentage of sites seems small compared to the large number of providers in the state, this level of reach and implementation is actually substantial. The technical assistance provided by OMH was shown to be crucial in achieving successful implementation and maintenance of CoCM.

New York is an extremely diverse state, with one of the biggest cities in the world and extremely rural areas. The heterogeneity of geography and population makes it challenging to design a uniform approach to engagement in any type of initiative statewide. The makeup of the healthcare system is equally varied, ranging from many single provider practices to numerous large, multi-specialty groups that are part of hospital networks with thousands of providers.

Other types of behavioral health integration programs exist in New York State, such as the primary care behavioral health (PCBH) model, which may seem similar to CoCM on the surface, but is actually quite different in its approach to integration ( [19, 20]. OMH took a more proactive role in encouraging primary care practices to adopt CoCM, not only providing TTA but also addressing regulatory and financing barriers through program design. For example, social workers in NYS are often unable to bill for services in primary care, but CCMP offers a revenue source that supports otherwise non-reimbursable components of integrated care, including care coordination and brief therapy delivered by social workers. OMH also argued strongly that clinics should be adopting evidence-based practices and focusing on outcomes, which CoCM does. This outcomes driven approach, along with the level of TTA provided at such a scale is unique in NYS. The advent of the CMS Final Rule that created the CoCM CPT codes for Medicare provided an external incentive for CoCM since NYS providers would be able to be reimbursed for a larger portion of their population, not only Medicaid patients. This likely improved the reach, adoption and implementation of CCMP by increasing the revenue opportunity and the potential for financial sustainability. Likewise, this impact may have been more significant for providers that were previously hesitant due to lower Medicaid populations.

The program was more successful among FQHCs compared to other primary care settings, reaching 83% of FQHCs and 3% of other primary care clinics. This is likely due to the fact that FQHCs have a very organized network which makes it easy to communicate and share information. NYS OMH presented at the state’s Community Health Center Association (CHCANYS) Conference on CCMP, and also provided webinars and other education. CHCANYS members often share resources and talk with one another, which led to many FQHCs reaching out to OMH after hearing about the successes of CCMP from another health center. Non-FQHC practices do not have a centralized network. They may or may not be a part of a hospital system, Independent Provider Association (IPA), or professional trade association, so it was difficult to ensure that the program was not missing providers. OMH did not have a specific target number of providers or practices to reach in CCMP, but did want to be sure that any interested providers were aware of the available support and financially sustainable opportunity. Increasing reach, especially among non-FQHC type clinics, is a high priority for the future. It may be interesting to look more closely at the characteristics of the practices that were reached but did not move forward to learn about what the barriers to adoption were and perhaps target outreach around those criteria.

This study demonstrates the importance of TTA and shows the effectiveness of high intensity, ongoing support in order to achieve sustained CoCM implementation. NYS OMH is fortunate to be able to provide high intensity TTA to a large number of practices. Each year, OMH has continued to modify the approach to TTA by using quarterly metrics data and surveys to assess the needs of clinics and through their feedback. TTA has become more individualized to address the unique challenges of NYS’s diverse practice population. This is especially true when it comes to support for billing. Helping clinics to be financially sustainable is a high priority for OMH, so training and coaching has become more focused on ensuring that practices are able to effectively bill for all payers. The data from this analysis reflects that billing was one of the biggest hurdles to implementation, with only 20% of those reached going on to bill Medicaid for services.

Previous literature suggests that even with a great deal of TTA geared toward implementing CoCM, NYS CCMP practices still needed post-implementation support to succeed in sustaining it [21]. OMH has used the data in clinics’ quarterly metrics reports to identify areas for quality improvement and reached out to specific clinics for targeted, post-implementation TTA. Though unable to assess intensity of TTA in relation to patient improvement rates due to the small number of clinics in each of the comparison groups, the relationship between high intensity TTA and successful implementation and sustainment of CoCM is strong. Improvement rates observed by clinics submitting at least one quarter of data matched those seen in previous RCTs studying the effectiveness of CoCM (45%) over routine care (19%), [1]. Recent studies have demonstrated the importance of TTA in ensuring successful implementation and outcomes. In addition, they found most real-world implementations of CoCM actually see outcomes below that of RCTs and those that NYS has achieved [21]. Future studies may take a deeper dive into the nuances of TTA such as the impact of different types, i.e., individual coaching calls vs. webinars or group trainings, and could also further define intensity.

Outcomes driven care is OMH’s ultimate goal for CCMP practices. OMH has focused support on implementation for the first few years of the program, but now is looking for ways to promote improved outcomes for practices that have longevity with CoCM. Going forward, they plan to use the quarterly metrics to provide performance feedback to sites and have already led small cohorts of clinics in Plan-Do-Study-Act cycles around increasing quarterly data accuracy in anticipation of this plan.

### Limitations

There were many limitations to the design and conduct of this study that restricted our ability to generalize from these data to other states and groups of clinics. First, this project was not designed as a study, but was a retrospective review of existing data, so the definitions of RE-AIM steps and their application was not completely consistent with the definitions as spelled out in the RE-AIM materials. The data collected were first primarily collected as administrative data. Not every variable was collected with analysis in mind. The work was done in New York State, which may be different in implementation culture and leadership from other states. Finally, this project was conducted during the same period as other New York behavioral health initiatives, such as the Delivery Systems Reform Incentive Payment (DSRIP) program and Thrive NYC. These initiatives had their own requirements for participation and data reporting, which may have impacted the outcomes of this study at any or all of the RE-AIM framework steps.

## Conclusion

Despite these limitations, there are many findings which might be important outside this study. First, it is possible to deliver a program at the state level and achieve broad representation of reach and adoption, at least geographically. OMH has increased the emphasis on technical assistance for billing activities in order to provide more support and ensure financial sustainability across all payers since the Medicare Final Rule was published in 2018. Future research efforts should focus on moving clinics from step to step, in order to increase acceptance and implementation over time. This first effort to step progress at the state level, toward meeting the goal of having all primary care settings in a defined area use principles of integrated care to treat depression, was lofty and aspirational, but these data give us hope that it can be done over time and with concentrated effort.

## Data Availability

The datasets used and/or analyzed during the current study are available from the corresponding author on reasonable request. Public access to the database(s) is closed.

## Abbreviations

CCMP: Collaborative Care Medicaid Program
CHCANYS: Community Health Center Association of New York State
CMS: Centers for Medicare and Medicaid Services
CoCM: Collaborative Care Model
DSRIP: Delivery System Reform Incentive Payment
FQHC: Federally Qualified Health Center
HRSA: Health Resources and Services Administration
IPA: Independent Provider Association
NYC DOHMH: New York City Department of Health and Mental Hygiene
NYS: New York State
OMH: Office of Mental Health
PCBH: Primary Care Behavioral Health Model
RCT: Randomized Control Trial
RE-AIM: Reach, Effectiveness, Adoption, Implementation, and Maintenance
TTA: Training and Technical Assistance

## References

[1] Archer J, Bower P, Gilbody S, Lovell K, Richards D, Gask L, et al. Collaborative care for depression and anxiety problems. Cochrane Common Mental Disorders Group, editor. Cochrane Database Syst Rev [Internet]. 2012 [cited 2020 Jan 7]; Available from: 10.1002/14651858.CD006525.pub2. 10.1002/14651858.CD006525.pub2 PMC11627142 23076925

[2] Gilbody S, Bower P, Fletcher J, Richards D, Sutton AJ (2006). Collaborative care for depression: a cumulative meta-analysis and review of longer-term outcomes. Arch Intern Med.

[3] Bower P, Gilbody S, Richards D, Fletcher J, Sutton A (2006). Collaborative care for depression in primary care. Making sense of a complex intervention: systematic review and meta-regression. Br J Psychiatry J Ment Sci.

[4] Thota AB, Sipe TA, Byard GJ, Zometa CS, Hahn RA, McKnight-Eily LR, Chapman DP, Abraido-Lanza AF, Pearson JL, Anderson CW, Gelenberg AJ, Hennessy KD, Duffy FF, Vernon-Smiley ME, Nease de Jr, Williams SP, Community Preventive Services Task Force (2012). Collaborative care to improve the management of depressive disorders: a community guide systematic review and meta-analysis. Am J Prev Med.

[5] Unutzer J, Katon W, Williams JW, Callahan CM, Harpole L, Hunkeler EM (2001). Improving primary care for depression in late life: the design of a multicenter randomized trial. Med Care.

[6] Girard A, Ellefsen E, Roberge P, Carrier JD, Hudon C (2019). Challenges of adopting the role of care manager when implementing the collaborative care model for people with common mental illnesses: a scoping review. Int J Ment Health Nurs.

[7] Bauer AM, Williams MD, Ratzliff A, Unützer J (2019). Best Practices for Systematic Case Review in Collaborative Care. Psychiatr Serv Wash DC.

[8] Sederer LI, Derman M, Carruthers J, Wall M (2016). The New York state collaborative Care initiative: 2012–2014. Psychiatr Q.

[9] Sederer LI (2014). What does it take for primary care practices to truly deliver behavioral health care?. JAMA Psychiatry.

[10] Glasgow RE, Vogt TM, Boles SM (1999). Evaluating the public health impact of health promotion interventions: the RE-AIM framework. Am J Public Health.

[11] DATA DICTIONARY NEW YORK STATE DEPARTMENT OF HEALTH. Provider Network Data System (PNDS) VERSION 10 (August 2020) [internet]. New York State Department of Health; Available from: https://www.health.ny.gov/health_care/managed_care/docs/dictionary.pdf.

[12] Collaborative Care: A step-by-step guide to implementing the core model [Internet]. Available from: https://aims.uw.edu/sites/default/files/CollaborativeCareImplementationGuide.pdf.

[13] Gellis ZD, Kenaley B (2008). Problem-solving therapy for depression in adults: a systematic review. Res Soc Work Pract.

[14] Care Management Tracking System (CMTS) [Internet]. Available from: https://aims.uw.edu/resource-library/care-management-tracking-system-cmts.

[15] Collaborative Care Medicaid Program Billing Application [Internet]. Available from: http://aims.uw.edu/nyscc/apply.

[16] 2019 New York State Collaborative Care Metrics [Internet]. Available from: https://aims.uw.edu/nyscc/sites/default/files/NYSCollabCare%20Metrics%202018%20FINAL.pdf.

[17] New York Health Center Data [Internet]. Available from: https://data.hrsa.gov/tools/data-reporting/program-data/state/NY.

[18] IBM SPSS statistics for Windows. Version 27. Armonk: IBM Corp.; 2019.

[19] Hunter CL, Funderburk JS, Polaha J, Bauman D, Goodie JL, Hunter CM (2018). Primary Care behavioral health (PCBH) model research: current state of the science and a call to action. J Clin Psychol Med Settings.

[20] Butz MR, Rynan WD (2020). Integrating Behavioral Healthcare and Primary Care, Appropriate Balance on What Model is Driving Care, and, the Whole Spectrum of Individuals are Coming Through the Door. J Clin Psychol Med Settings.

[21] Moise N, Shah RN, Essock S, Jones A, Carruthers J, Handley MA (2018). Sustainability of collaborative care management for depression in primary care settings with academic affiliations across New York state. Implement Sci.

